# Effectiveness of Two Dietary Approaches on the Quality of Life and Gastrointestinal Symptoms of Individuals with Irritable Bowel Syndrome

**DOI:** 10.3390/jcm9010125

**Published:** 2020-01-02

**Authors:** Maria Margarida Guerreiro, Zélia Santos, Elisabete Carolino, Julieta Correa, Marilia Cravo, Fátima Augusto, Cristina Chagas, Catarina Sousa Guerreiro

**Affiliations:** 1 Universidade de Lisboa, Faculdade de Medicina, Laboratório de Nutrição, 1649-045 Lisboa, Portugal; margarida.guerreiro@live.com.pt (M.M.G.); j.nunes.correa@gmail.com (J.C.); 2 Escola Superior de Tecnologia da Saúde, Instituto Politécnico de Lisboa, 1990-096 Lisboa, Portugal; etcarolino@estesl.ipl.pt; 3 Hospital de Egas Moniz, Centro Hospitalar de Lisboa Ocidental, 1349-019 Lisboa, Portugal; zelia.coelho.santos@gmail.com (Z.S.); cchagas@chlo.min-saude.pt (C.C.); 4 Hospital Beatriz Ângelo, Serviço de Gastrenterologia, 2674-514 Loures, Portugal; marilia.cravo@sapo.pt; 5 Hospital da Luz Setúbal – Serviço de Gastrenterologia, 2900-722 Setúbal, Portugal; fnaugusto63@gmail.com

**Keywords:** irritable bowel syndrome, food, low fodmap diet, symptoms, quality of life

## Abstract

To evaluate the effectiveness of a low FODMAP (fermentable oligosaccharides, monosaccharaides, disaccharides and polyols) diet in the relief of symptoms and an improvement of the quality of life in individuals with irritable bowel syndrome in comparison to a standard diet according to the British Dietetic Association’s guidelines. A non-randomized clinical trial of adult patients with IBS was compared two diet interventions. An assessment of symptoms, quality of life, and nutritional status was performed before and after the four-week mark of intervention. Individuals from the Low FODMAP Diet (LFD) group were evaluated on a third moment, after the controlled reintroduction of FODMAPs. A total of 70 individuals were divided in two groups: Low FODMAP Diet (LFD; *n* = 47) and Standard Diet (SD; *n* = 23). 57 individuals completed the four-week intervention (LFD; *n* = 39; SD; *n* = 18). At the completion of four weeks, the symptoms improved in both groups (LFD: *p* < 0.01; DC: *p* < 0.05) but LFD led to a higher relief (*p* < 0.05), primarily with respect to abdominal pain and diarrhoea. Quality of life improved significantly in both groups, with no significant differences between SD vs LFD (*p* > 0.05). In the LFD group, the relief of symptoms observed at the four-week mark remained constant after reintroduction of FODMAPs. Both interventions seem to be effective for the relief of symptoms and quality of life, however LFD had higher effectiveness in the former. The results with LFD suggest it can be a preferred approach in individuals with diarrhoeal profile.

## 1. Introduction

Irritable bowel syndrome (IBS) is a chronic functional gastrointestinal disorder with a complex and multifactorial aetiology that can be explained by mechanisms including changes in the nervous system pathways in connection with the gut, dysbiosis, altered gut permeability, genetics, among other physio/physiological factors and/or of social nature [1]. Dietary therapy is considered a first line of treatment for IBS patients [2,3]. In recent years, short-chain carbohydrates have increasingly been pointed out as nutrients that can potentially impact the onset of symptoms characteristic of IBS [4,5,6,7,8,9]. FODMAPs (fermentable oligosaccharides, monosaccharaides, disaccharides and polyols) have a slow and incomplete absorption in the small intestine, contributing to an increase of the osmotic gradient in the lumen and lead to bacterial fermentation that are part of intestinal microbiota [10]. A diet rich in FODMAPs leads to an increased presence of water and gas in the intestinal lumen, which in combination with other factors (of physiological, psychosocial nature and/or from an environmental source) can trigger symptomatology characteristic of an IBS diagnosis [10,11]. 

This evidence supports a low FODMAP diet compounds as an effective dietary approach to IBS treatment. Despite recent evidence, some aspects of this diet are still controversial, namely its efficiency when compared to a standard diet, maintenance in a medium/long term and its nutritional efficacy [11]. However, studies performed in populations of Mediterranean origin are scarce. The present study, performed in a population of Mediterranean origin, suggests a low FODMAPs diet as a therapeutic approach, supported by the assessment of its effectiveness in improving symptoms and quality of life of individuals with IBS. 

## 2. Materials and Methods

### 2.1. Participants

This multicentre sample comprised outpatients followed at Egas Moniz Hospital, Beatriz Angelo Hospital and Luz Setubal Hospital. The selected individuals were adults (18–80 years old) diagnosed with IBS in accordance with the criteria of Rome IV. The following criteria were considered for exclusion: diagnosis of other gastrointestinal diseases; presence of severe comorbidities or previous clinical situations (ex.: abdominal surgery) that might influence the gastrointestinal symptoms; use of supplements pre and/or probiotics during the study; use of antibiotics during the study and/or in the previous four weeks; significantly restrictive dietary pattern before the beginning of the study (ex.: total exclusion of gluten; vegan; low fodmap diet). The prior exclusion of lactose was accepted, provided that it is kept constant during the intervention period. All participants received written and oral information about the study, before signing an Informed Consent. The Ethics Committee of Centro Hospitalar Lisboa Ocidental approved the protocol for the present study.

### 2.2. Study Design

#### 2.2.1. Moment 1: Baseline Evaluation

At first, all participants went through nutritional assessment. The protocol was explained to each participant, including that it would comprise a comparison and analysis of two distinct dietary approaches. Sociodemographic and clinical data were collected, and an assessment of symptoms and quality of life performed. Individuals were then divided in two study groups: Low FODMAP Diet and Standard Diet, and attributed a personalized dietary plan taking into consideration macro and micronutrients as well as distinct dietary guidelines distinct for each of the two groups.

At the initial moment, the study protocol was explained to each participant, and they were asked to participate in a study that would test the efficacy of two distinct dietary approaches in the improvement of IBS symptoms. Patients who fulfilled the entry criteria were randomized in a 2:1 ratio to low FODMAP diet vs Standard Diet for IBS, as they were referred to nutrition appointment. This was a diet trial, and therefore the dietitian was not blinded. However the authors that analyzed the data were blinded to randomization. Sociodemographic and clinical data were collected, while dietary pattern, GI symptoms, and quality of life were also evaluated. Each group of participants received a personalized eating plan with appropriate macro and micronutrients levels, with distinct dietary recommendations for each group.

#### 2.2.2. Moment 2: 4-Week Follow-Up

After four weeks of dietary intervention, all individuals were re-assessed using the same parameters as those used in the first assessment. The participants of the LFD group completed a questionnaire that assessed their satisfaction of the dietary intervention.

#### 2.2.3. Moment 3: LFDs 10-Week Follow-Up

**Figure 1:**
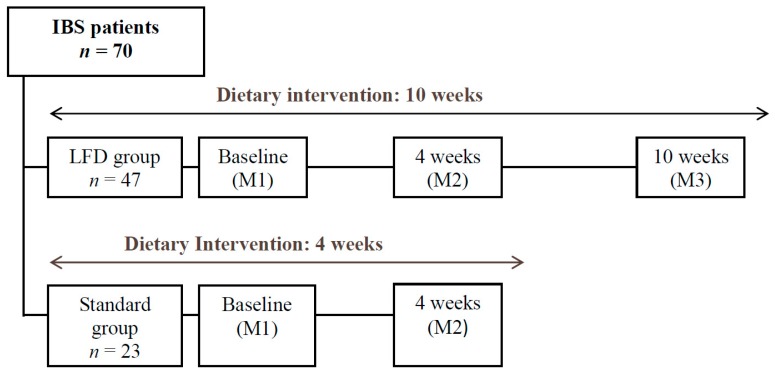
Study Design; M, Moment of Evaluation.

Only individuals from the LFD Group were requested to be present at this final evaluation, with the purpose of analyzing the results of their diet in the medium term, after the controlled reintroduction of foods high in FODMAPs (Figure 1).

### 2.3. Dietary Approaches Studied 

The Low FODMAP Diet is divided in two phases: exclusion phase (4 weeks) and reintroduction phase (6 weeks). Initially, the subjects were instructed to exclude all foods rich in FODMAPs over a 4-week period, which includes for example: wheat, rye and barley foods; legumes; mushrooms; sweet potatoes; cabbage; garlic; onions; some fruits as mango and figs; honey and lactose foods. As an alternative, some Low FODMAP foods that may be included at this stage include: rice, oats, carrots, courgettes, tomatoes, oranges, pineapple; strawberries and lactose-free products. As supporting material, in addition to the food plan, each participant received a manual with a brief explanation of the principles of the diet, a list of foods to avoid, and respective food alternatives. At the reintroduction phase, subjects were instructed to perform a controlled introduction of “test-foods”, rich in each class of FODMAPs, in order to assess the individual’s tolerance. Each “test-food” was introduced on its own, with increasing portions over a period of three days. A record for progression of symptoms was also requested.

The Standard Diet was based on first-line dietary recommendations from National Institute for Health and Care Excellence (NICE) guidelines, namely: small and regular meals during the day; monitoring intake of alcohol and drinks containing caffeine; monitoring the intake of meals and/or foods with high fat content; monitoring intake of foods high in fibre, especially insoluble fibre; increasing water and other non-sweetened fluids intake; test tolerance to lactose, which implied a period of exclusion when necessary.

To evaluate the symptoms the following scales were used: Birmingham IBS Symptom Score Questionnaire (BISS), for the frequency of gastrointestinal symptoms [12]; visual analog scale (VAS), to assess the severity of each of the 10 symptoms: abdominal pain, abdominal distension, malaise/nausea, constipation, diarrhea, heartburn, fatigue, depression, anxiety [13]; Dichotomic question “Do you consider that your symptoms were adequately controlled in the last 7 days?”, for the adequate relief of symptoms [14].

The quality of life assessment was performed using the Irritable Bowel Syndrome - Quality of Life Questionnaire (IBS-QOL) composed of 34 questions that measure the quality of life specifically for IBS patients [15]. 

### 2.4. Assessment of the Dietary Intake and Nutritional Status 

Food intake was analyzed using the 24-hour dietary recall method, in all of the three moments previously described. The quantitative analysis of nutritional intake and FODMAPs was carried out with the support of a food quantification manual [16] and tables with FODMAP nutritional content of each food. A measurement of body weight, body mass index, waist circumference, and body composition (through bipolar electrical bioimpedance, Tanita BC-351) was performed at all moments of assessment.

### 2.5. Statistical Analysis 

A statistical analysis was performed with Statistical Package for the Social Science software (SPSS Inc., version 21, IBM Company, Chicago, IL, USA). A comparison of variables in the same group (baseline vs follow-up) was performed using a T-test for two paired samples or the Wilcoxon test. The comparison of results between groups (LFD vs DC) was performed with using a T-test for two independent samples or the Mann-Whitney test. For the inferential statistical measures, we considered a level of significance for *p* < 0.05. 

## 3. Results

### 3.1. Participants

Seventy individuals were selected for the study (74.3% women; 65.7% professionally active, average age: 48.5 ± 14.7 years) and were divided in two groups: Low FODMAP diet group (*n* = 47) and Group Standard Diet (*n* = 23).

Thirteen individuals abandoned the study in the first four weeks of intervention, 8 in LFD Group and 5 in SD Group, due to the impossibility of keeping the dietary recommendations (*n* = 3), absence from the country (*n* = 2) and for other reasons, not specified (*n* = 8). In the second phase of assessment 57 individuals were included, 39 in the LFD group and 18 in the SD group.

Thirty-two individuals completed the intervention of 10 weeks in LFD group. Seven individuals abandoned the study during the reintroduction phase due to not being able to access the hospital (*n* = 3), opting out from completing the intervention (*n* = 1) or for other reasons, not specified (*n* = 3). The present study included individuals with all subtypes of IBS (Table 1). 

### 3.2. Gastrointestinal Symptoms (Week 4)

The overall score for the frequency of symptoms decreased significantly in both groups compared to baseline (LFD: *p* < 0.001; SD: *p* < 0.05). However, the amplitude of the reduction was higher in the LFD Group (*p* = 0.041). (Table 2) With respect to specific symptoms, the LFD proved to be more effective in relation to the SD in reducing pain and diarrhoea. Although the SD led to a reduction in the frequency of constipation, the difference between the diets was not statistically significant. The general classification of the VAS significantly decreased for both groups compared to the baseline (LFD: *p* < 0.001; SD: *p* < 0.05). However, the amplitude of the difference was greater in the LFD group (*p* = 0.005). (Table 2) The LFD led to a significant reduction in the intensity of symptoms for all the assessed individuals.

To calculate the rate of overall success of dietary interventions, we only considered individuals who have changed to a positive response when asked the question “Do you consider that your symptoms were adequately controlled in the last seven days?”, between the first and second moments of evaluation. The success rate of the LFD in general improvement of symptoms was 56.4%, significantly higher than the 22.2% success rate observed in SD group (*p* = 0.016).

### 3.3. Quality of Life (week 4)

The overall score for quality of life increased significantly in both groups compared to the baseline (LFD: *p* < 0.001; SD: *p* < 0.05), with no statistically significant difference between groups (*p* = 0.227). (Table 2) LFD led to a significant reduction of the impact of the IBS at the following areas of quality of life, covered in IBS-QOL: dysphoria, concern with health, interference in daily activities, body image, sexual life, social life and relationships with others. Furthermore, the LFD group showed a greater proportion of individuals with an improvement of the QOL score (M1-M2 > 14) in relation to the DC group, despite the non-statistically significant difference (*p* = 0.084). 

### 3.4. Nutritional Status (Week 4) 

At the completion of four weeks following the given dietary recommendations, we observed a significant decrease of energy and carbohydrates intake in both groups. Comparing the two dietary approaches, the intake of dietary fibre and iron was significantly lower in the LFD (Table 3).

Both groups showed a significant decrease in the total intake of FODMAPs for four weeks, however, this decrease was significantly higher in the LFD Group (Table 3).

### 3.5. Anthropometry (Week 4) 

Individuals of the LFD group showed a significant decrease in weight, body mass index, abdominal circumference, and percentage of fat mass after four weeks of exclusion of FODMAPs. Comparing results between the two dietary interventions, the decrease of weight and decreased BMI was more evident in the LFD group (Table 4).

### 3.6. Adherence and Satisfaction (Week 4)

In the LFD group, the rate of adherence to four weeks (83.0%) was slightly higher than the SD group (78.3%). The rate of satisfaction concerning the exclusion phase of the LFD was 79.1%, covering areas such as the ease of integration of nutritional guidelines in the daily routine, food palatability and usefulness of the given support materials. The outlay was the domain that presented a lower rate of satisfaction.

### 3.7. Low FODMAP Diet: medium-term evaluation (Week 10)

Once the reintroduction phase of foods rich in FODMAPs was concluded, individuals of the LFD Group were evaluated at a third moment, completing 10 weeks since the beginning of the intervention. At this point, 68.1% (*n* = 32) of the LFD group completed the three moments of evaluation where no significant differences were found when compared to the second assessment. This comparison was made based on symptoms, quality of life and nutritional intake (*p* > 0.05). (Table 5).

## 4. Discussion

Our findings suggest that the exclusion of foods rich in FODMAPs can lead to a significant improvement of IBS symptoms, with higher impact when compared to the standard diet. Supported by other authors, we found the LFD is effective in controlling symptoms of IBS, especially at the level of pain, abdominal distension and diarrhea [4,17,18,19,20,21].

By including individuals with all subtypes of IBS, namely diarrheic, constipated, and mixed, our study allowed us to compare the effectiveness of the LFD in individuals with distinct symptoms alongside their respective clinical results.

As observed in previous studies, the LFD was less effective in the control of constipation [3,17,21,22,23]. On the contrary, in case of diarrhea, the restriction of FODMAPs leads to a decreased osmolarity and, consequently, decreased water content in the intestinal lumen, which in turn becomes an advantageous consequence for this subtype of IBS. LFD can also be effective in the reduction of intraluminal fermentation and consequent control of symptoms such as pain and abdominal distension, frequently present in any subtype of IBS. Therefore, the choice of the dietary approach should always be individualized, according to several factors.

No differences were previously observed in the efficacy of the approaches regarding quality of life, with both leading to a significant improvement in the overall score [17,18,19,24,25]. However, our study showed higher effectiveness for LFD in improving individuals QOL according to the IBS-QOL scores. At 10 weeks, although no statistically significant difference compared to the second moment of assessment, the overall score of QOL kept a positive progression. Even though the improvement of symptoms is swiftly noticeable, this may reflect a more gradual change in everyday life and in the attitude of the individuals towards himself and those who surround them. This study fails to assess the long-term QOL, which may be a better indicator of the real impact of the interventions, as well as addressing the possible change of effectiveness of the given diets. 

For the duration of the initial four weeks, and as expected, the ingestion of all classes of FODMAPs was significantly reduced in the LFD group, supporting this diet as a sustainable diet with a good adherence level and effective nutritional approach model (via coaching/advice and support material). Both diets in this study led to a significant reduction of the intake of energy and carbohydrates for four weeks, results often observed in similar studies [3,4,21,25]. Although the energy restriction has not been the object of our intervention, it may be a consequence from following a personalized dietary plan. Furthermore, both dietary approaches led to a significant reduction in body weight and body mass index. Considering the high prevalence of overweightness in the initial sample, the given results can be seen as a positive indicator. The observed weight loss, reduced abdominal perimeter, and decrease in the fat mass index at the four-week mark was more accentuated in the LFD Group, possibly due to the more restrictive nature of this approach. At 10 weeks, the anthropometric parameters for the LFD group stabilized, suggesting the absence of nutritional risk increased in the long-term dietary approach.

Both diets did not alter significantly the fibre intake, calcium, potassium, and magnesium. However, the intake of these nutrients was below the DRI from the time of initial evaluation. The intake of fibre and iron was lower for individuals who were following the LFD. The lower intake of overall fibre in the LFD Group should be considered as an explanatory hypothesis of higher symptom improvement, rather than just the restriction of FODMAPs. In the medium and long term, regardless of the chosen dietary approach, further nutritional assessments should take place in order to improve the intake of fibre and overall micronutrients (e.g., fortified foods and/or supplementation). Additionally, in LFDs the reintroduction phase should give room for a detailed assessment of the individual tolerance to each food allowing a greater choice of foods while in remission to prevent any nutritional deficiency and potentially higher adherence.

However, our investigation is limited by the sample size and for not being a blind clinical trial. The compliance to the dietary recommendations was totally dependent on the adherence of the participants, since they were not provided with meals. However, our intervention was carried out in a context similar to the real clinical practice, including individuals with all subtypes of IBS and based on a personalized diet therapy and nutritional advice. Additionally, both diets discussed in our study (LFD and SD) were initially developed as nutritionally suitable to control and manage gastrointestinal symptoms. When compared to other studies that evaluate the effectiveness of LFD, they have failed in doing so by recurring to conventional diet patterns or diets high in FODMAPs to establish the same comparison. On a last note, the duration of our intervention (10 weeks) was higher than the average of the studies previously published (around four weeks).

In summary, this study brings forward the LFD as an effective dietary approach in the control of symptoms and improvement of the quality of life in individuals with IBS. Compared to conventional dietary recommendations, an LFD proves to be an advantage in the management of IBS symptoms, especially in individuals with diarrheic profile. Our results support the use of LFD in a practical clinical context, potentially implying higher rates of adherence and patient satisfaction.

## 5. Conclusions

Both diets appear to be effective in IBS therapy. However, the LFD proved a higher efficacy in the relief of symptoms and ha a superior positive impact in most of the domains of quality of life, therefore it could be a dietetic approach to favor in IBS therapy, mainly in individuals with diarrheic subtype.

## Figures and Tables

**Table 1:** Baseline characteristics.

Clinical and Demographics Variables	Low FODMAP Diet (*n* = 39)	Standard Diet (*n* = 18)	*p* Value
Age, mean ± SD	49.5 ± 14.0	52.3 ± 17.3	ns
Female, *n* (%)	32 (82.1)	12 (66.7)	ns
Professionally active	30 (77)	8 (55.6)	ns
BMI, mean ± SD	25.4 ± 4.6	27.4 ± 4.0	ns
BISS, mean ± SD	19.1 ± 7.9	16.1 ± 7.3	ns
VAS-Total, mean ± SD	50.9 ± 19.9	43.6 ± 22.3	ns
VAS-Pain, mean ± SD	6.0 ± 2.5	4.2 ± 2.9	0.024
IBS Subtype			ns
IBS-D, *n* (%)	15 (38.5)	9 (50.0)	
IBS-C, *n* (%)	11 (28.2)	6 (33.3)	
IBS-M, *n* (%)	13 (33.3)	3 (16.7)	

BMI, Body Mass Index; BISS, Birmingham IBS Symptom Score; IBS, Irritable Bowel Syndrome; VAS, Visual Analogue Scale; IBS-D, Irritable Bowel Syndrome Diarrhoea Subtype; IBS-C, Irritable Bowel Syndrome Constipations Subtype; IBS-M, Irritable Bowel Syndrome Mixed Subtype; QOL, Quality of Life; ns, Not Significant.

**Table 2:** Symptoms and Quality of Life Evolution.

IBS Scores	LFD			SD			*p* **
	M1	M2	*p* *	M1	M2	*p* *	M2-M1
BISS Total	19.1	10.8	0.000	16.1	12.1	0.036	0.041
BISS-Pain	7.7	3.3	0.000	6.6	4.9	ns	0.005
BISS-Diarrhoea	6.5	3.4	0.000	5.4	4.6	ns	0.025
BISS-Constipation	5.0	4.1	ns	4.1	2.6	0.041	ns
VAS Total	50.9	28.8	0.000	43.5	37.0	0.013	0.005
IBS-QOL Total	61.1	70.9	0.000	64.5	70.6	0.032	ns

Data are mean value; *p* * evolution within group (M1 vs. M2); *p* ** comparison of the evolution (M1 vs. M2) between groups; BISS, Birmingham IBS Symptom Score; VAS, Visual Analogue Scale; IBS, Irritable Bowel Syndrome; QOL, Quality of Life; M, Moment of Evaluation. IBS-QOL was calculated with the formula: 100 − ((sum of the items − lowest possible score / raw score range) × 100), with higher values indicating better QOL.

**Table 3:** Nutritional Intake.

Energy and Nutrients	LFD			DC			*p* ** (M1)	*p* ** (M2)
	M1	M2	*p* *	M1	M2	*p* *		
Energy (kcal/d)	1785	1650	0.001	1932	1697	0.006	ns	ns
Protein (g/d)	77.3	80.1	ns	74.2	72.5	ns	ns	ns
Fat (g/d)	64.9	57.5	ns	68.0	61.1	ns	ns	ns
CHO (g/d)	212.0	191.6	0.02	236.0	204.2	0.016	ns	ns
Fibre (g/d)	20.6	18.5	ns	21.4	21.7	ns	ns	0.048
Calcium (mg/d)	586.3	609.3	ns	621.7	612.0	ns	ns	ns
Magnesium (mg/d)	239.5	219.1	ns	254.4	232.0	ns	ns	ns
Iron (mg/d)	9.3	6.7	0.000	10.3	8.9	ns	ns	0.003
Potassium (mg/d)	2645.7	2677.6	ns	2647.0	2657.7	ns	ns	ns
FODMAPs (g/d)	13.0	3.9	0.000	15.0	10.3	0.012	ns	0.000
Lactose	3.9	0.2	0.000	5.5	3.4	ns	-	-
Fructose	3.0	1.2	0.005	3.3	0.8	ns	-	-
FOS	3.9	1.2	0.000	3.9	3.8	ns	-	-
GOS	0.4	0.2	0.000	0.5	0.6	ns	-	-
Polyols	1.9	1.1	0.008	1.7	1.6	ns	-	-

Data are mean value; *p* * evolution within group (M1 vs. M2); *p* ** comparison between groups; CHO, carbohydrates; FODMAPs, Fermentable Oligosaccharides, Disaccharides, Monosaccharides and Polyols; FOS, Fructooligosaccharides; GOS, Galactooligosaccharides.

**Table 4:** Anthropometric measures and body composition.

Nutritional Variables	LFD			SD			*p* ** M2-M1
	M1	M2	*p* *	M1	M2	*p* *	
Weight (kg)	65.6	64.6	0.000	74.0	73.8	ns	0.014
BMI (kg/m^2^)	25.4	25.0	0.000	27.4	27.1	ns	0.049
WC (cm)	88.7	86.1	0.000	94.1	92.4	0.006	ns
FM (%)	30.8	30.0	0.034	33.7	33.9	ns	ns

Data are mean value; *p* * evolution within group (M1 vs. M2); *p* ** comparison of the evolution (M1vs. M2) between groups; BMI, Body Mass Index; WC, Waist Circumference; FM, Fat Mass.

**Table 5:** LFD Group: 10-week evaluation.

Variable	M2	M3	*p* *
BISS			
Total	10.3	10,6	ns
BISS-Pain	3.0	3,7	ns
BISS-Diarrhea	3.5	3,5	ns
BISS-Constipation	3.8	3,4	ns
VAS Total	27.3	30,0	ns
IBS-QOL Total	72.3	76.1	ns
Energy (kcal/d)	1666	1692	ns
CHO (g/d)	194.0	197.3	ns
Iron (mg/d)	6.8	6.7	ns
Fibre (mg/d)	18,4	20,0	ns
BMI (kg/m^2^)	25.4	25.3	ns

Data are mean value; *p* * evolution within group (M2 vs. M3); BISS, Birmingham IBS Symptom Score; VAS, Visual Analogue Scale; IBS, Irritable Bowel Syndrome; QOL, Quality of Life; CHO, Carbohydrates; BMI, Body Mass Index.
